# A method to detect fulvestrant interference in estradiol in breast cancer patients

**DOI:** 10.1530/EC-23-0178

**Published:** 2023-10-03

**Authors:** Margarida Brito, Susana Prazeres, Marta Malheiros

**Affiliations:** 1 Department of Medical Oncology, Instituto Português de Oncologia de Lisboa Francisco Gentil, Lisboa, Portugal; 2 Department of Clinical Pathology, Instituto Português de Oncologia de Lisboa Francisco Gentil, Lisboa, Portugal

**Keywords:** fulvestrant, estradiol, immunoassay, breast cancer

## Abstract

**Background:**

Fulvestrant resembles estradiol in its structure. Reports have been published concerning fulvestrant measured as estradiol by the immunoassays. This may induce falsely elevated estradiol results and wrongly impact medical decisions. Our aim was to confirm the interference of fulvestrant on estradiol concentration and test a method to identify the false results.

**Methods:**

Four serum samples with low estradiol levels were spiked with fulvestrant at various concentrations. Estradiol was then measured directly on serum (Dir), after a 1:5 dilution (Dil), and a ratio Dil/Dir was estimated. On the second part of the study, estradiol results (Dir, Dil and ratio Dil/Dir) from 14 women treated with fulvestrant were analysed, as well as from 14 patients not under this treatment.

**Results:**

The addition of exogenous fulvestrant to the serum samples induced a gradual rise on estradiol concentration with a mean ratio for the Dil/Dir samples of 2.1 ± 0.4 (range 1.7–2.9). Patients on fulvestrant treatment experienced a mean ratio for the Dil/Dir estradiol sample of 2.4 ± 0.4 (range 1.6–3.0). In the control group, a mean estradiol ratio Dil/Dir of 1.1 ± 0.1 was observed (range 0.8–1.3). No correlation between the number of days after fulvestrant injection and estradiol result (*r* = 0.531) was observed.

**Conclusion:**

Our study confirmed the interference of fulvestrant in the estradiol measurement by immunoassay. When fulvestrant was present, the estradiol ratio for Dil/Dir sample was about 2. In the control group, the ratio was around 1. The estradiol Dil/Dir ratio is a simple tool which can be used to identify fulvestrant false immunoassay estradiol results.

## Introduction

Fulvestrant was the first selective estrogen receptor degrader (SERD) (1) to enter clinical practice and represents, alone or in combination with targeted therapies, a key compound for endocrine advanced breast cancer (ABC). As a drug, it competes with estradiol with high affinity to the estrogen receptor (ER); the complex fulvestrant–ER causes an unstable conformational change with ER degradation and inhibition of estrogen signalling (2).

Fulvestrant was mainly studied in postmenopausal patients (3). Yet, the recent positive results from the trials PALOMA 3 (4) and MONARCH 2 (5) in the ABC premenopausal groups ER+ HER2− have come to support its generalized use in these patients in association with CDK4/6 inhibitors and also in monotherapy.

In premenopausal patients, fulvestrant requires the combination with a gonadotropin‐releasing hormone (GnRH) agonist therapy for ovarian suppression (OS) (6). However, studies show that 17–24% (7, 8) of premenopausal women do not experience complete OS with GnRH agonists therapy and, although still not clear, this may contribute to poorer outcomes (7, 9, 10). Some authors (7, 8) suggest that estradiol measurement should routinely be carried out in these patients to ensure that they are indeed fully castrated.

**Figure 1:**
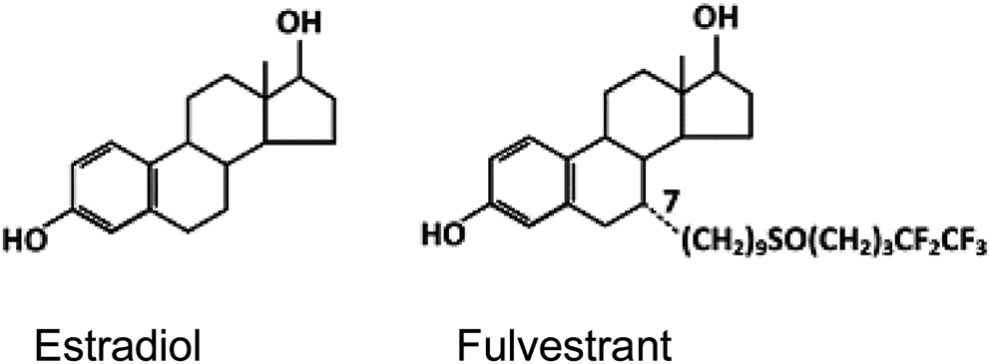
Chemical structure similarity between estradiol and fulvestrant.

Fulvestrant resembles estradiol in its molecular structure (Fig. 1) leading to a cross-reactivity in estradiol immunoassays (11). A few case reports (12, 13, 14) have been published concerning fulvestrant measured as estradiol by the commercial immunoassays and inducing falsely elevated estradiol results. This is a problem for clinicians and laboratory staff and may wrongly impact medical decisions.

Our aim was to confirm the interference of fulvestrant on estradiol concentration and test a simple laboratory method to identify the false results.

## Methods

### Study design

This was a single-center, observational study with retrospective data collection, separated into two parts. Initially, four samples of serum with low estradiol levels, obtained from our laboratory serum bank, were spiked with fulvestrant at various concentrations. A serum sample with low estradiol concentration (16.5 pg/mL, sample 1) was aliquoted and spiked with fulvestrant 200 ng/mL to obtain the final concentrations of 4, 8, 12, 16, 20, 24, 28 and 32 ng/mL. These concentrations were chosen because, after administration, fulvestrant is slowly absorbed and maximum plasma concentrations are reached after approximately 5 days. At steady state, fulvestrant plasma concentrations are between 16.3 and 25.1 ng/mL (15). The three remaining serum samples had respectively estradiol concentrations of 22.3 pg/mL (sample 2), 40.1 pg/mL (sample 3) and 56.1 pg/mL (sample 4) and were also aliquoted and spiked with fulvestrant 200 ng/mL to get the concentrations of 8 and 20 ng/mL. Estradiol was measured on each aliquot, directly (Dir) and, to confirm results, after a 1:5 onboard dilution (Dil) with estradiol diluent (the 1:5 dilution factor was already pre-defined by the Centaur CP system assay for estradiol). To evaluate the proportion between the two measurements, a ratio Dil/Dir was also estimated.

On the second part of the study, we reviewed the medical records and analysed the estradiol results (Dir, Dil and ratio Dil/Dir) from 14 women with stage IV hormone-receptor-positive HER 2 negative advanced breast cancer treated with fulvestrant in our center. The estradiol levels of 14 female patients not under this treatment, previously measured for routine in our laboratory, were used as a control group.

The study was approved by our Institutional Ethics Committee. Due to the retrospective nature of the study, and because adequate measures to protect data confidentiality were provisioned, permission to waive informed consent was given.

### Fulvestrant

A pre-filled syringe of commercial fulvestrant (250 mg/5 mL, EVER Valinject/Kent Pharma, Heathrow, UK) was used to prepare a solution of 200 ng/mL in ethanol (Merck).

### Estradiol measurement assay

Estradiol was measured by a competitive chemiluminescent immunoassay (Enhanced Estradiol, Centaur CP, Siemens Healthcare Diagnostics Inc, Tarrytown, NY, USA). The test range is 10.7–3000 pg/mL, the limit of blank is 7.9 pg/mL and the limit of detection is 10.7 pg/mL. The intra- and inter-assay precisions are 9.2% and 4.5%, respectively, at a concentration of 39.3 pg/mL. The reference interval for considering post-menopausal females cutoff is <32.2 pg/mL (16). The definition for complete ovarian suppression while on a GnRH agonist is not clearly defined (8). We used the estradiol cut-off levels for naturally postmenopausal women.

### Statistical analysis

Data were analysed as mean ± s.d. and range. The Pearson correlation coefficient was used to evaluate the correlation between the number of days after fulvestrant injection and estradiol level.

## Results

The addition of fulvestrant (0–32 ng/mL) to sample 1 induced a gradual rise in estradiol concentration, from 16.5 pg/mL (basal) to 930 pg/mL, corresponding to an increase of 5636%. A similar behavior was observed for samples 2, 3, and 4 (Table 1). In the spiked samples, the estradiol level of the 1:5 diluted sample, compared with the direct one, is about two times higher. The mean estradiol ratio obtained for the Dil/Dir samples is 2.1 ± 0.4 with a 1.7–2.9 range.

**Table 1:** Estradiol concentration of the serum samples spiked with fulvestrant.

Fulvestrant concentration	Estradiol (pg/mL)											
	Sample 1			Sample 2			Sample 3			Sample 4		
	Dir	Dil	Ratio Dil/Dir	Dir	Dil	Ratio Dil/Dir	Dir	Dil	Ratio Dil/Dir	Dir	Dil	Ratio Dil/Dir
0 ng/mL	16.5			22.3			40.1			56.1		
4 ng/mL	49.3	137	2.8									
8 ng/mL	101	219	2.2	146	423	2.9	190	411	2.2	194	391	2.0
12 ng/mL	152	354	2.3									
16 ng/mL	231	560	2.4									
20 ng/mL	522	1006	1.9	526	1017	1.9	686	1241	1.8	803	1326	1.7
24 ng/mL	495	1025	2.1									
28 ng/mL	689	1348	2.0									
32 ng/mL	930	1641	1.8									

Dil, 1:5 dilution; Dir, direct.

In the second step of the study, we analysed 27 estradiol samples (Dir, Dil and ratio Dil/Dir) from 14 patients under fulvestrant therapy (patients 1 to 14). The days between fulvestrant injection and blood collection as well as the estradiol level prior fulvestrant therapy are shown in Table 2, when available. The patients under fulvestrant therapy had an estradiol level between 116 and 838 pg/mL. The estradiol ratio Dil/Dir ranged from 1.6 to 3.0 with a mean value of 2.4 ± 0.4. Prior to the beginning of fulvestrant treatment, these women exhibited a low estradiol concentration (≤46 pg/mL). We observed no correlation between the number of days after fulvestrant injection and estradiol result (*r* = 0.531). The data are summarized in Table 2.

**Table 2:** Estradiol concentration of serum samples from patients under fulvestrant therapy.

Patient	Age (years)	Estradiol (pg/mL) pre-Fulvestrant	Days after fulvestrant injection	Estradiol Dir (pg/mL)	Estradiol Dil (pg/mL)	Ratio Dil/Dir	Treatment	Time on fulvestrant (months)^a^
1	80	–	8	116	302	2.6	Fulvestrant	0.3
2	55	17.0	28	149	373	2.5	Fulvestrant + Palbociclib	5
3	48	–	27	161	299	1.9	Fulvestrant + Palbociclib	7
4	49	46.0	32	204	524	2.6	Fulvestrant + Palbociclib + Goserelin	27
5	48	–	29	210	366	1.7	Fulvestrant + Palbociclib	24
			1	608	1814	3.0		30
6	60	–	27	214	478	2.2	Fulvestrant + Palbociclib	5
7	52	33.0	30	226	536	2.4	Fulvestrant + Palbociclib	16
			27	306	778	2.5		17
			13	224	604	2.7		18
8	49	29.0	28	239	592	2.5	Fulvestrant + Goserelin	44
			28	301	757	2.5		46
			28	364	817	2.2		48
			28	389	999	2.6		50
			28	248	581	2.3		52
			30	317	805	2.5		54
9	54	<20	22	252	664	2.6	Fulvestrant + Palbociclib	25
			21	298	744	2.5		26
			21	187	569	3.0		27
10	45	12.6	NA	313	677	2.2	Fulvestrant + Goserelin	5
11	43	–	31	452	1168	2.6	Fulvestrant + Goserelin	13
12	55	<10.7	19	457	799	1.7	Fulvestrant	2
13	48	<10.7	6	498	863	1.7	Fulvestrant + Palbociclib	0,2
			15	277	432	1.6		1
14	49	–	7	838	1892	2.3	Fulvestrant + Ribociclib	48
			14	534	1253	2.3		50
			28	233	634	2.7		53

For patients with more than one estradiol measurement, the results are presented chronologically.

^a^Time since the beginning of fulvestrant until the blood collection. Dil, 1:5 dilution; Dir, direct; NA, not available.

In the group of patients not under fulvestrant (controls 1 to 14), the estradiol concentration was between 98 and 1444 pg/mL with a mean ratio Dil/Dir of 1.1 ± 0.1 (range 0.8–1.3) (Table 3).

**Table 3:** Estradiol concentration of serum samples from the control group (patients not under fulvestrant therapy).

Control	Age (years)	Estradiol Dir (pg/mL)	Estradiol Dil (pg/mL)	Ratio Dil/Dir	Patient clinical information
1	31	98	112	1.1	Breast cancer stage II, ER+, HER2−, under adjuvant tamoxifen + goserelin.
2	49	102	108	1.1	Breast cancer stage I, ER+, HER2+, under adjuvant exemestane.
3	50	114	152	1.3	Breast cancer stage I, ER+, HER2−, under adjuvant tamoxifen.
4	52	157	173	1.1	Breast cancer stage II, ER**−**, HER2**−**, on follow-up.
5	26	210	223	1.1	Acute myeloid leukemia at 12 years old underwent allotransplant, on follow-up.
6	19	221	182	0.8	Borderline ovarian left tumour at 18 years old, performed surgery, on follow-up.
7	45	279	246	0.9	Neurofibromatosis type I on follow-up.
8	46	313	370	1.2	Breast cancer stage III, ER**−**, HER2+, on follow-up.
9	52	369	425	1.2	Breast cancer stage I, ER+, HER2+, under adjuvant tamoxifen.
10	47	378	420	1.1	Breast cancer stage III, ER+, HER2+, under adjuvant tamoxifen.
11	51	480	532	1.1	Breast cancer stage II, ER+, HER 2+, under adjuvant tamoxifen.
12	46	660	659	1.0	Breast cancer stage II, ER+, HER2**−**, under adjuvant tamoxifen.
13	45	1041	1002	1.0	Breast cancer stage II, ER+, HER2+ under adjuvant tamoxifen.
14	32	1444	1531	1.1	Hodgkin lymphoma at 25 years old, performed chemotherapy protocol ABVD and autologous transplant, on follow-up.

Dir, direct; Dil ,1:5 dilution; ER, estrogen receptor; Protocol ABVD, doxorubicin, bleomycin, vinblastine and dacarbazine.

## Discussion

The chemical structure similarity between estradiol and fulvestrant makes it impossible for the antibodies used in the immunoassays to distinguish among them (15, 16). Consequently, an increase in estradiol level due to cross-reaction with fulvestrant is observed (12, 14). In our study, we confirmed the interference of fulvestrant in the estradiol measurement by Centaur CP immunoassay and proposed a simple laboratory tool that allows to differentiate false cases from actual ovarian escape. The increase in serum estradiol level after the addition of exogenous fulvestrant showed that it was measured as estradiol in the used immunoassay. An alternative type of analysis to measure estradiol with no anticipated cross-reactivity to fulvestrant such as liquid chromatography-mass spectrometry could be used and has been recommended (15). However, it is not a practical method for a routine clinical laboratory.

The fulvestrant half-life is estimated to be 50 days (15, 17) remaining in circulation for a longer period than the interval between administrations (28 days). This is an additional problem since the interference caused by this drug may persists for months (14, 18).

The estimation of the estradiol ratio between the diluted and the direct serum sample (ratio Dil/Dir) permitted to identify the interference of fulvestrant on estradiol quantification, by immunoassay. In the serum where fulvestrant is present, we found that the estradiol ratio for Dil/Dir sample is about 2. In the serum of patients not under fulvestrant, this ratio is around 1. Fulvestrant forms spontaneous and reversible colloidal aggregates in an aqueous solution, which can be converted to the monomeric form by dilution (19, 20, 21). Moreover, 99% of fulvestrant binds, reversibly, to plasma proteins (mainly very low, low- and high-density lipoprotein fractions) (15, 22). The fulvestrant feature of colloidal aggregates formation and its bind to plasma proteins can explain why, after sera dilution and consequent disaggregation, the measured estradiol increases in samples with the drug, leading to a ratio Dil/Dir of about 2.

The study results are also meaningful for clinical practice. Since efficacy data in premenopausal women became available (4, 5), fulvestrant (with GnRH for ovarian suppression) in combination with CDK4/6 or in monotherapy is a widely used option in ABC premenopausal women ER+. Failure to attain complete ovarian suppression with GnRH can however occur (7, 8) and may conceivably be associated with higher recurrence risk (9, 10). So, although not a level I recommendation, routine monitorization of estradiol in these patients may be considered. Patients on fulvestrant with chemotherapy-induced amenorrhea and no GnRH support, especially when younger, may experience ovarian recovery initially without bleeding and can also be candidates to estradiol measurements. Assuming an ovarian escape in a patient, and not being aware of the fulvestrant falsely elevated estradiol levels and how to distinguish it, can create errors and mistakenly influence medical decisions. It may result in unneeded surgical castration, unnecessary interruption of fulvestrant, an increase in blood tests and costs, and inconvenience for the patient and the clinician.

In our center, we also observed unexpectedly high estradiol levels not consistent with the patient’s clinical history. This triggered the discussion and the study resulted from a straight collaboration between the clinical and the laboratory team, emphasizing once again the critical role of multidisciplinary work and communication in the management of cancer patients.

In conclusion, we present the estimation of the estradiol Dil/Dir ratio as a simple tool to detect fulvestrant interference and identify the false Centaur CP immunoassay estradiol results.

## Declaration of interest

The authors declare that there is no conflict of interest that could be perceived as prejudicing the impartiality of the research reported.

## Funding

This research did not receive any specific grant from any funding agency in the public, commercial or not-for-profit sector.

## Ethics committee approval

The study was approved by our Institutional Ethics Committee. Due to the retrospective nature of the study, and because adequate measures to protect data confidentiality were provisioned, permission to waive informed consent was given. The ethics committee is Instituto Português de Oncologia Francisco Gentil Lisboa Ethics Committee for Health (e-mail: comissaoetica@ipolisboa.min-saude.pt).

## Data availability statement

The data underlying this article are available in the article.

## Author contribution statement

All authors have contributed equally to the study design, data collection, results interpretation, draft and revision of the manuscript and approval of the final version. All authors agreed to be accountable for all aspects of the work in ensuring that questions related to the accuracy or integrity of any part of the work were appropriately investigated and resolved.
